# Overexpression of the tonoplast sugar transporter CmTST2 in melon fruit increases sugar accumulation

**DOI:** 10.1093/jxb/erx440

**Published:** 2017-12-23

**Authors:** Jintao Cheng, Suying Wen, Shuang Xiao, Baiyi Lu, Mingru Ma, Zhilong Bie

**Affiliations:** College of Horticulture and Forestry, Huazhong Agricultural University and Key Laboratory of Horticultural Plant Biology, Ministry of Education, Wuhan, P. R. China

**Keywords:** *Cucumis melo*, strawberry, fruit, sugar accumulation, sugar transporter, tonoplast, TST2, vacuolar

## Abstract

Fruits are an important part of the human diet and sugar content is a major criterion used to evaluate fruit quality. Melon fruit accumulate high sugar concentrations during their development; however, the mechanism through which these sugars are transported into the vacuoles of fruit cells for storage remains unclear. In this study, three tonoplast sugar transporters (TSTs), CmTST1, CmTST2, and CmTST3, were isolated from melon plants. Analysis of subcellular localization revealed that all these proteins were targeted to the tonoplast, and evaluation of spatial expression and promoter-GUS activity indicated that they had different expression patterns in the plant. RT-PCR and qRT-PCR results indicated that CmTST2 exhibited the highest expression level among the TST isoforms during melon fruit development. Histochemical and immunohistochemistry localization experiments were performed to identify the tissue- and cell-type localization of CmTST2 in the fruit, and the results indicated that both its transcription and translation were in the mesocarp and vascular cells. Overexpressing the *CmTST2* gene in strawberry fruit and cucumber plants by transient expression and stable transformation, respectively, both increased sucrose, fructose, and glucose accumulation in the fruit. The results indicate that CmTST2 plays an important role in sugar accumulation in melon fruit.

## Teaser

CmTST2 is a tonoplast sugar transporter that is highly expressed during melon fruit development, and its overexpression also increases sugar accumulation in strawberry and cucumber fruits.

## Introduction

Sugars in plants are synthesized in mesophyll cells and translocated to the other parts of the plant, particularly sink organs, where they were used to supply the carbon substrate for growth and/or storage. In most plants, sucrose is directly loaded into the phloem by two types of sugar transporters: SWEET proteins, which export sucrose out of leaf cells (Chen *et al.*, 2010, 2012) and sucrose transporters (SUTs/SUCs), which mediate sucrose into phloem cells with protons (Lalonde *et al.*, 2004). In sinks that accumulate soluble sugars to high concentrations, such as fleshy fruit including apples (Zhang *et al.*, 2004), grapes (Zhang *et al.*, 2006), and tomatoes (Ruan and Patrick, 1995), sucrose unloading may involve an apoplasmic step. The unloaded sucrose may be absorbed by adjacent recipient sink cells across the plasma membrane through SUTs/SUCs (Weber *et al.*, 1997) or hexose transporters (STP/HT) and undergo hydrolysis by cell wall invertase (CWIN) (Ruan *et al.*, 2010). Upon entering the cell, a major fraction of the sugars is stored in the vacuole, which occupies up to 90% of the plant cell volume. The transport of sucrose and glucose across the vacuole (by either proton antiporter activity or facilitated diffusion) has been demonstrated at the molecular level. Members of three sugar transporter families can mediate sugars across the vacuole; these include tonoplast monosaccharide transporters (TMTs) (Wormit *et al.*, 2006) and vacuolar glucose transporters (VGTs) (Aluri and Büttner, 2007) from the monosaccharide transporter family; some of SUC4-type sucrose transporters from the sucrose transporter (SUCs/SUTs) family (Reinders *et al.*, 2008; Eom *et al.*, 2011; Schulz *et al.*, 2011; Schneider *et al.*, 2012); and there members (AtSWEET2, 16, and 17) of the SWEET protein family (Chardon *et al.*, 2013; Klemens *et al.*, 2013; Guo *et al.*, 2014; Chen *et al.*, 2015). However, amongst all the transporters that have been identified, only one member from the TMTs subfamily, namely BvTST2.1 isolated from sugar beet, has been identified in sugar storage organs; this protein acts as a sucrose proton antiporter and is responsible for vacuolar sucrose uptake in sugar beet taproots (Jung *et al.*, 2015). To date, the type of sugar transporters involved in other sugar-accumulating organs, such as fleshy fruits, remains unclear.

Melon (*Cucumis melo*) is a typical plant that can transport raffinose family oligosaccharides (RFO), particularly stachyose and raffinose, in the phloem (Bachmann *et al.*, 1994; Dai *et al.*, 2006; McCaskill and Turgeon, 2007). Melon fruit do not have polysaccharides but instead accumulate high concentrations of sucrose at the late stage of development (Lingle and Dunlap, 1987; Mitchell *et al.*, 1992). The accumulation of sucrose in melon fruit is more complicated than sucrose transport in plants. The developmental process is regulated and determined by the metabolism of carbohydrates in the fruit sink itself (Hubbard *et al.*, 1989; Lester *et al.*, 2001). Melon fruit undergo a metabolic transition from the early stage of growth to sucrose accumulation, which is primarily by loss of activity of soluble acid invertase and up-regulation of two genes (*CmSPS1* and *CmSPP1*) (Lester *et al.*, 2001; Burger and Schaffer, 2007). To date, most studies on sugar metabolism and accumulation in melon fruit have focused on the activities of a few enzymes, such as cell wall acid invertase and sucrose phosphate synthase (SPS) (Hubbard *et al.*, 1989; Lester *et al.*, 2001; Burger and Schaffer, 2007). However, the mechanism by which sugars, especially sucrose, are transported into the vacuoles from the cytosol and accumulate to high concentrations in the fruit has not yet been determined.

In this study, we isolated and identified three *TST* genes (*CmTST1*, *CmTST2*, and *CmTST3*) from melon plants. The subcellular localization of *CmTST*-GFP fusion proteins was examined in tobacco epidermal cells and protoplasts. Detailed expression patterns during plant development were analysed by expressing the *CmTST*-promoter-GUS complex in Arabidopsis. qRT-PCR analysis was also performed in different organs of melon plants, and the expression patterns during fruit development and sugar accumulation were assessed through RT-PCR and qRT-PCR analyses. *CmTST2* was found to be the most highly expressed TST isoform during melon fruit development as sugar accumulates. The tissue- and cell-type localization of CmTST2 was evaluated by histochemical analysis of transiently transformed melon fruit expressing the *CmTST2*-promoter-GUS fusion. Immunohistochemistry localization experiments were also conducted. The *CmTST2*-overexpression construct was transformed into strawberry fruit and cucumber plants to identify the physiological roles of CmTST2 in plants.

## Materials and methods

### Plant materials

Melon (*Cucumis melo* L., cv. Yilishabai; cv. Yangjiaosu; cv. Xiaomaisu) and cucumber (*Cucumis sativus* L., wild-type cv. Xintaimici, and transgenic lines) plants were grown in polytunnels from March to June or from July to November. Tissues were sampled to test for gene transcription and sugar quantification. Different tissues (root, stem, leaf, petiole, female flower, male flower, and fruit) were collected from 2-month-old plants and used for spatial expression. Melon fruit at different developmental stages between 0–40 d after anthesis (DAA) were also harvested for temporal expression and sugar quantification. All tissues and fruit samples were immediately frozen in liquid nitrogen and stored at −80 °C. Strawberry (*Fragaria × ananassa* Duch., cv. Falandi) plants were grown in a polytunnel during spring seasons from 2016 to 2017 in Wuhan (Midland China).

*Arabidopsis thaliana* ecotype Col-0 was grown in potting soil (Sunshine® Mix #1 Fafard^®^-1P; www.sungro.com) or on agar medium in a growth chamber under short-day (8 h light/16 h dark) or long-day (16 h light/8 h dark) conditions at 22 °C and 55% relative humidity. Arabidopsis transformation was performed with *Agrobacterium tumefaciens* strain GV3101 (Holsters *et al.*, 1980) by using the floral dip method (Clough and Bent, 1998).

### Subcellular localization

Subcellular localization was evaluated using the method described by Sugiyama *et al.* (2017) with some modifications. To analyse the subcellular localization of CmTST1/2/3 in *Nicotiana benthamiana*, we generated *CmTST1*/*2*/*3*-GFP (green fluorescent protein) fusion constructs by using the vector pH7LIC5.0-ccdB rc-N-eGFP using the primers listed in Supplementary Table S1 at *JXB* online. *Agrobacterium tumefaciens* GV3101 was transformed with an expression clone carrying the *CmTST1*, *CmTST2*, or *CmTST3* coding sequence (CDS) N-terminus fused with eGFP under the 35S promoter. Bacterial cultures were grown in YEB medium at 28 °C overnight. The cultures were harvested and resuspended in infiltration buffer (10 mM MES, pH 5.6, 10 mM MgCl_2_, and 200 mM acetosyringone) to an OD_600_ cell density of approximately 1.0. The post-transcriptional gene-silencing suppressor p19 was co-agroinfiltrated to enhance the expression of GFP or RFP (Kim *et al.*, 2007). For co-expression, two cultures carrying appropriate constructs and the p19 cultures were mixed in a 1:1:0.5 ratio to OD_600_=0.4:0.4:0.2. The mixture was incubated at room temperature for 1–3 h and infiltrated into the lower surface of 5–6-week-old *N. benthamiana* leaves by using a 1-ml syringe. At 3 d after infiltration, the agroinfiltrated *N. benthamiana* leaves were cut into small pieces and mounted in water on glass slides for confocal microscopic analysis. The fluorescence was imaged using an FV1200 Olympus confocal microscope. The eGFP and mRFP1 fluorescence was excited using 488- and 584-nm laser lines, respectively. Protoplasts were prepared 3 d after infiltration and imaged using the same confocal microscope.

### Isolation of the promoter region and GUS expression analysis

The promoter sequences of *CmTST1* (1869 bp), *CmTST2* (1845 bp), and *CmTST3* (1978 bp) were isolated through amplification of genomic DNA using the primers listed in Supplementary Table S1. The PCR products were recombined to the pBI121 vector, upstream of the GUS (β-glucuronidase) gene, to obtain p*CmTST1-GUS*, p*CmTST2-GUS*, and p*CmTST3-GUS* fusion constructs. These constructs were transformed into col-0 wild-type Arabidopsis plants by using *A. tumefaciens* strain GV3101 and the floral dip method (Clough and Bent, 1998). The p*CmTST1-GUS* construct was also transformed into sections of melon fruit *in vitro* by using the transient expression method (Cheng *et al.*, 2015*a*). Briefly, the fresh melon fruit were cross-cut into 0.5–1-cm thick sections and incubated in Murashige and Skoog (MS) liquid medium (containing *A. tumefaciens* cells harboring p*CmTST1-GUS*) under vacuum (1.5 atm) for 20 min at 28 °C. After removing the surface liquid, the sections were placed on MS solid medium and cultured at 28 °C in darkness for 24–36 h. Prior to staining, the sections were cleaned using distilled water containing 500 mg l^−1^ carbenicillin. For GUS staining, the transgenic Arabidopsis samples or the melon sections were incubated with GUS staining solution overnight at 37 °C using the method described by Jefferson *et al.* (1987). After staining, the green tissues were dehydrated through an ethanol series and photographed.

### Cloning of CmTSTs

To determine the putative tonoplast sugar transporters that are responsible for sugar accumulation in melon fruit, we used the nucleotide sequence of *BvTST2.1* (sugar beet taproot tonoplast sucrose transporter 2.1, gene bank accession no. XM_010680330.2) as queries for a BLAST search of the translated melon genome (http://www.ncbi.nlm.nih.gov/BLAST/;Madden *et al.*, 1996). Only three putative CmTSTs, showing high identity at the protein level with BvTST2.1, were obtained and were designated as CmTST1, CmTST2, and CmTST3. The open reading frames (ORFs) of *CmTST1*, *CmTST2*, and *CmTST3* were isolated by RT-PCR from melon fruit (see Supplementary Table S1 for the primers used) and the sequences were separately cloned into pGEM-T easy vectors (Promega, USA) and sequenced.

### RT-PCR and qRT- PCR

Total RNA was extracted from specific tissues by using TRIzol according to the manufacturer’s instructions (http://www.transgen.com.cn/). The RNA was reversely transcribed using SuperScript III reverse transcriptase (Invitrogen, USA) with RNase H (Invitrogen, USA). The cDNA was used as template for RT-PCR or qRT-PCR analysis. For RT-PCR analysis, the gene-specific primers used are listed in Supplementary Table S1, and the 18S ribosomal RNA was used as a control. qRT-PCR was performed on the ABI7500 system (Bio-Rad) using the SYBR green detection protocol (TaKaRa). Primers used for qRT-PCR analysis are given in Supplementary Table S1. Finally, the mean expression level of relevant genes was calculated using the 2^−ΔΔ^Ct method (Livak and Schmittgen, 2001).

### Determination of soluble sugar

Collected samples stored at –80 °C were ground to powder with liquid nitrogen for sugar determination. The pulverized tissues (1–2 g) were ultrasonically extracted three times in 7–8 ml of 80% (v/v) ice-cold methanol at 70 °C for 30 min. The supernatant was pooled and mixed with an internal standard (methyl-a-D-glucopyranoside, Sigma, USA). The mixture was dried using Speed Vac (Eppendorf, Hamburg, Germany) and derivatized using hydroxylamine hydrochloride: hexamethyldisilazane (HMDS): tri methyl chloro silane (TMCS). The derivatized sample extracts were analysed by GC-MS (Agilent 7890B) with a HP-5 MS column by using previously described methods (Bartolozzi *et al.*, 1997; Rojas-Escudero *et al.*, 2004) with some modifications. Sugars were quantified with internal standards by using the following retention times: sucrose, 15.804 min; glucose, 11.439 and 11.593 min; and fructose, 11.034 and 11.102 min.

### Generation of anti-CmTST2 antisera and immunolocalization of CmTST2 in melon fruit

Two specific peptide fragments (AGNDSDDNLRSPLIS and KWSEREGPDGNKEGGFK) derived from the CmTST2 protein sequence were selected to synthesize polypeptides and used for immunization of two rabbits (specific pathogen-free) by AtaGenix. To test the quality of the antisera, membrane proteins from yeast cells expressing *CmTST1*, *CmTST2*, *CmTST3*, or empty pDR196 vector were prepared, and 200 μg of each preparation was separated on SDS-PAGE, blotted onto nitrocellulose, and incubated with anti-CmTST2 antiserum. For immunohistochemical analysis, melon fruit at 0 DAA were cut into transverse paraffin sections (14 μm) and treated as previously described (Cheng *et al.*, 2015*a*). The specimens were viewed with a Nikon Eclipse 80i microscope.

### Construction of expression vector and transfection in strawberry fruit by agroinfiltration

To construct the *CmTST2* overexpression vector, we isolated the coding region of *CmTST2* from Yilishabai melon fruit using a PCR method. The DNA was digested with XbaI /SmaI and cloned into the PBI121 vector under the control of the CaMV *35S* promoter. Then the vector was transformed into *A. tumefaciens* LBA4404. The primers used are listed in Supplementary Table S1.

The vector was transfected into strawberry fruit by using the method described by Jia *et al.* (2013) with some modifications. *Agrobacterium tumefaciens* was grown in YEB liquid medium (containing 10 mM Mes, 20 mM acetosyringone, 50 mM antibiotic) at 28 °C overnight to an OD_600_ of approximately 1.0. The cultures were harvested and resuspended in infiltration buffer (10 mM MgCl_2_, 10 mM Mes, pH 5.6, and 200 mM acetosyringone) to an OD_600_ of approximately 0.8 and kept for 2 h at room temperature before being used for infiltration. Strawberry fruit at the late green stage were injected while they were still attached to the plants by using a sterile 1-ml hypodermic syringe. A marker pen was used to divide one fruit on each plant into two halves. One half was injected with a carefully controlled volume of the *Agrobacterium* suspension carrying the *CmTST2* overexpression vector such that it was sufficient to infuse just that half of the fruit. The other half was injected with the *Agrobacterium* suspension carrying the PBI121 empty vector. All the injected fruit were elevated so that they were held up above the plants and hence received uniform light.

### Generation of transgenic cucumber plants

For the generation of transgenic cucumber, *A. tumefaciens* with the *CmTST2* overexpression vector as described above was used. The *CmTST2* overexpression vector was transformed into the cucumber pure line Xintaimici by using the fresh expanding cotyledon disk transformation method as previously described (Cheng *et al.*, 2015*b*). The regenerated plants were screened by RT-PCR analysis.

### Statistical analysis

Student’s *t*-tests were performed using the algorithm embedded into Microsoft Excel, and significance was evaluated at the 5% level (*P*<0.05) for all comparisons. For each treatment, the standard error of the mean (SE) was calculated based on at least three biological replicates.

## Results

### Isolation and homological analysis of three TST genes from melon plants

**Fig. 1.**
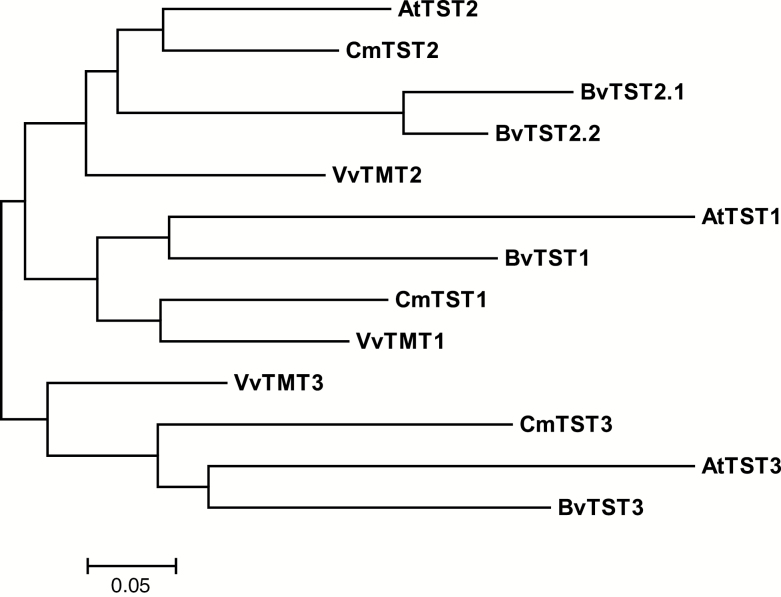
Phylogenetic tree analysis of CmTSTs. The dendrogram was constructed using MEGA version 6.0 (Tamura *et al.*, 2013) adopting Poisson correction distance using the bootstrap method with 1000 replicates, and it is presented as a traditional rectangular tree view. GenBank accession numbers and sources for the respective sequences are: CmTST1 (XP_008464819.1), CmTST2 (XP_008448165.1), and CmTST3 (XP_016899284.1) from melon; AtTMT1 (At1g20840), AtTMT2 (At4g35300), and AtTMT3 (At3g51490) from Arabidopsis; VvTMT1 (HQ323282.1), VvTMT2 (HQ323283.1), and VvTMT3 (HQ323284.1) from grape; BvTST1 (XP_010686712.1), BvTST2.1 (XP_010678631.1), BvTST2.2 (XP_010690557.1), and BvTST3 (XP_010680636.1) from sugar beet.

Basing on the publicly accessible *Cucumis melo* genome (Garcia-Mas *et al.*, 2012), we identified three TST orthologs, which are homologous to *AtTST1*/*2*/*3* (*AtTMT1*/*2*/*3*) in Arabidopsis. The sequences from the melon genome, with ORFs of 2190-, 2202-, and 2178-bp nucleotides, were designated as *CmTST1*, *CmTST2*, and *CmTST3* and encoded peptides of 729, 733, and 725 amino acids with calculated molecular weights of 78.4, 78.4, and 77.4 kDa, respectively. Phylogenetic analysis revealed that CmTST1, CmTST2, and CmTST3 were closely related to the Arabidopsis homologs AtTST1, AtTST2, and AtTST3, respectively (Fig. 1). CmTST2 shared approximately 81% amino acid identity with AtTST2 and the newly identified sucrose-specific transporter BvTST2.1 in sugar beet (see Supplementary Fig. S1). The hydrophobicity profile predicted that, similar to AtTMT1, CmTST2 contains two sets of six transmembrane domains separated by a uniquely large (328 amino acid residues in length) central hydrophilic loop (Supplementary Figs S1 and S2) (Wormit *et al.*, 2006). CmTST1 and CmTST3 also possess a remarkably long centrally located loop (Supplementary Fig. S2).

### CmTST2 is the most highly expressed TST isoform during melon fruit development

**Fig. 2.**
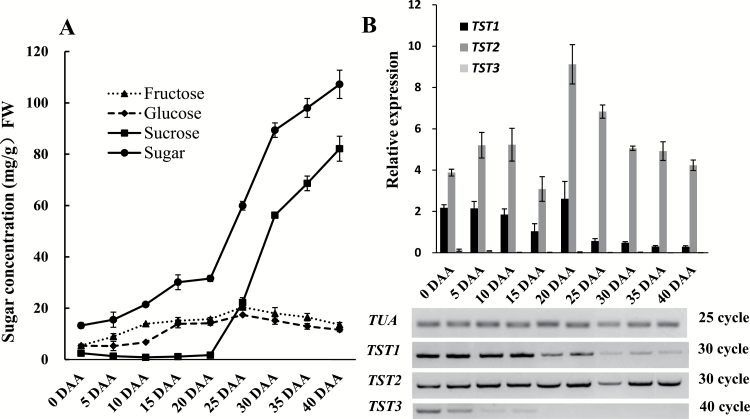
Relationship between sugar content and mRNA levels of three *CmTST* genes. (A) Contents of different types of sugar in melon fruit during different developmental stages. (B) mRNA levels of three *CmTST* genes in melon fruit during different developmental stages, as determined by RT-PCR and qRT-PCR. *TUA* was used as the reference gene. The number of PCR cycles and the band intensity represent the expression level of the genes: fewer cycles and stronger bands represent higher expression. Error bars represent the SE for three technical replicates of three biological replicates. DAA, days after anthesis.

To identify the most highly expressed TST isoform in melon fruit and to determine the putative relationship between its expression and sugar levels in the fruit, we evaluated different types of sugar content and the relative transcript levels of the three CmTST isoforms during fruit development. Little sucrose could be detected in the early stages of fruit development up to 20 DAA, after which it accumulated rapidly to high concentrations (about 80 mg g^–1^ FW) (Fig. 2A). Glucose and fructose mainly accumulated in the early stages up to 25 DAA and then remained at relatively low concentrations (about 15 mg g^–1^ FW) (Fig. 2A). qRT-PCR and RT-PCR analyses showed that *CmTST2* consistently exhibited the highest expression throughout the entire fruit development period, especially during the late stages (after 20 DAA). However, the expression of *CmTST1* was also high in the early stage of development, although it decreased quickly after 20 DAA. *CmTST3* was hardly detected throughout the entire course of fruit development, with only a weak band being visible in the young fruit when more PCR cycles were conducted (Fig. 2B). In addition, we also detected the expression of *CmTST2* in two other genotypic melon varieties, Xiaomaisu and Yangjiaosu, that accumulated much less sugar (especially sucrose) than the Yilishabai cultivar during fruit development (see Supplementary Fig. S3). Expression of *CmTST2* in the high sugar-containing variety Yilishabai were much higher than in the low sugar-containing varieties Xiaomaisu and Yangjiaosu (Supplementary Fig. S3B, C). Overall, the results indicated that *CmTST2* was the most highly expressed TST isoform during melon fruit development and therefore it may play an important role in sugar accumulation in the fruit.

### Subcellular localization of CmTST proteins

**Fig. 3.**
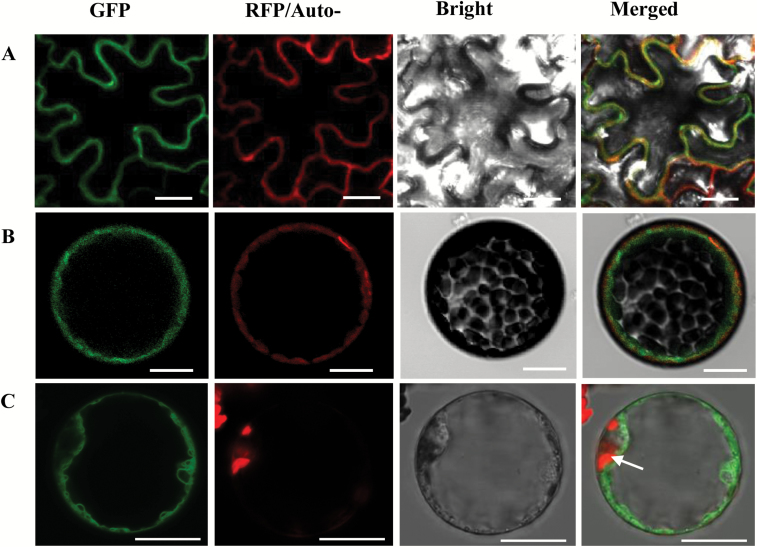
Subcellular localization of an N-terminal CmTST2–GFP fusion protein in tobacco. (A, B) Co-localization of CmTST2–GFP and GAMMA-tip:RFP (a tonoplast localization marker) in tobacco epidermal cells (A) and protoplast (B). (C) Tonoplast localization of CmTST2–GFP in tobacco protoplasts. The laser-scanning confocal microscopy images show fluorescence and merged images. Chlorophyll auto fluorescence (Auto-) in (C) and the bright-field images are also presented. The CmTST2–GFP fusion protein was clearly inserted into the tonoplast, which separated the vacuolar volume from the cytoplasm containing the chloroplasts (white arrow). Scale bars =20 μm.

To determine the subcellular localization of CmTST, we generated constructs encoding *CmTST1-GFP*, *CmTST2-GFP*, and *CmTST3-GFP* under the control of the cauliflower mosaic virus 35S promoter. Each construct was co-expressed with the mRFP1-labeled tonoplast marker (Gamma-tip, Arabidopsis tonoplast aquaporin) in *N. benthamiana* leaves by infiltration with agrobacteria. Confocal images of *Agrobacterium*-infiltrated *N. benthamiana* leaves demonstrated that CmTST2 was co-located with the tonoplast marker to the tonoplast (Fig. 3A). Protoplasts were prepared from the infiltrated leaves for further observation, and the results indicated that CmTST2-GFP and GAMMA-tip:RFP were clearly co-located to the tonoplast of the tobacco protoplast (Fig. 3B). Furthermore, as shown in Fig. 3C, CmTST2-GFP fluorescence was clearly distinct from chloroplast autofluorescence and it revealed localization of the fusion protein to the tonoplast and not the plasma membrane. For CmTST2-GFP, the fusion proteins CmTST1-GFP and CmTST3-GFP were also located in the vacuolar membrane, as shown by expressing them in tobacco epidermal cells and protoplasts (see Supplementary Fig. S4). These results strongly suggest that CmTST1, CmTST2, and CmTST3 are localized at the tonoplasts and are not targeted to the plasma membrane.

### Expression analysis of CmTST1, CmTST2, and CmTST3 in different tissues

To examine the tissue-specific gene expression of *CmTST1*, *CmTST2*, and *CmTST3*, we performed qRT-PCR and RT-PCR analyses on different tissues of melon plants. The results revealed that *CmTST1* and *CmTST2* were highly expressed in the roots, stem, leaf, petioles, female flowers, and male flowers in addition to the fruit tissues (see Supplementary Fig. S5). On the other hand, CmTST3 expression was not detected in any tissue; after running the PCR with more cycles, only a weak band was detected in the male flower (Supplementary Fig. S5B).

**Fig. 4.**
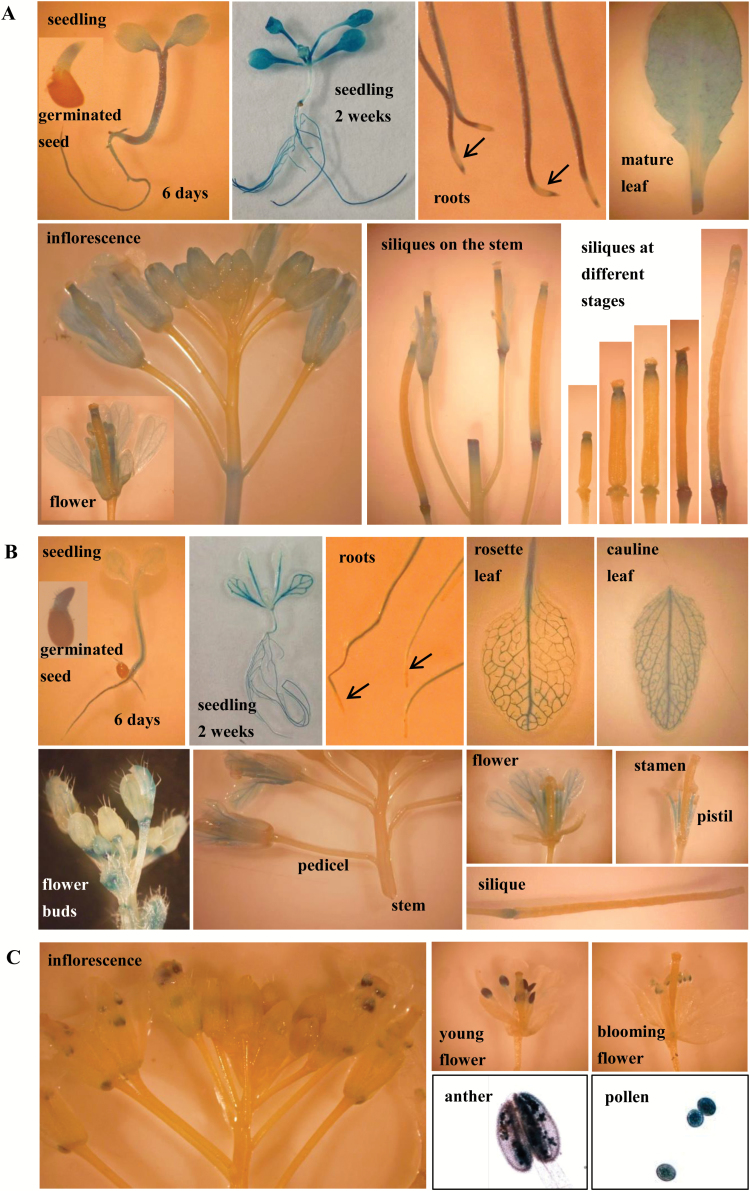
Expression of the GUS reporter gene in Arabidopsis plants under the control of the *CmTST* promoter. (A) *CmTST1*-promoter-GUS; (B) *CmTST2*-promoter-GUS; (C) *CmTST3*-promoter-GUS. (This figure is available in colour at *JXB* online.)

To confirm the expression patterns of the three CmTSTs and to obtain detailed expression profiles during plant development at the tissue level, we generated transgenic Arabidopsis plants that expressed the GUS gene under the control of the *CmTST1*, *CmTST2*, or *CmTST3* promoters. As shown in Fig. 4A, the *CmTST1* gene was actively transcribed in different tissues. Strong GUS activity was detected in the roots and hypocotyl of 6-d-old seedlings, whereas only slight staining was observed in the radicle and cotyledon. In 2-week-old seedlings, strong GUS staining was detected in all leaf and root samples. Detailed examination showed that the GUS staining did not cover the entire root; specifically, there was no staining in the elongation zone. GUS staining covered all the leaf vein and mesophyll tissues in mature leaves. In the inflorescence stem, GUS staining was detected in the sepals, petals, and stigma/style of almost all developing flower buds. Mature flowers showed the presence of GUS staining in the filament and anther envelope but not in the pollen (Fig. 4A, the pollen image is not shown). During development of the siliques, GUS staining seemed to shift from the stigma/style to the abscission zone. Otherwise, GUS activity under the control of the *CmTST1* promoter was extremely high in all the abscission and wound sites, as shown in the leaf and stem.

As shown in Fig. 4B, *CmTST2*-promoter-GUS activity was mainly restricted to the vascular tissues, as observed in the cotyledon, hypocotyl, juvenile leaves, mature leaves (rosette and cauline leaves), sepals from flower buds, and petals from mature flowers. We also detected GUS staining in emerging radicles, roots, filaments, and the abscission zone of flowering leaves and siliques, in addition to the vascular tissues. Similar to *CmTST1*, GUS staining did not cover all the roots and was absent in the root tip.

As shown in Fig. 4C, *CmTST3*-promoter-GUS activity was restricted to the pollen grains, which is consistent with the results from PCR (see Supplementary Fig. S5B). Moreover, the putative cis-elements in the promoter regions of *CmTST1-3* were also predicted from the PlantCARE (Lescot *et al.*, 2002; http://bioinformatics.psb.ugent.be/webtools/plantcare/html/) and PLACE (Higo *et al.*, 1999; http://www.dna.affrc.go.jp/htdocs/PLACE/) databases. A number of tissue-/organ-specific relative elements, including leaf and root high-expression relative elements, were found in the promoters of *CmTST1* and *CmTST2* (Supplementary Table S2). The putative promoter of *CsTST3* contained several pollen-specific motifs (Supplementary Table S2). These results were also consistent with those of the promoter-GUS activity analysis. Overall, the results indicated that *CmTST3* is a pollen-specific gene whilst *CmTST1* and *CmTST2* exhibit varied expression levels depending on tissue type and developmental stage.

### Histochemical and immunohistochemical analyses of CmTST2 in melon fruit

**Fig. 5.**
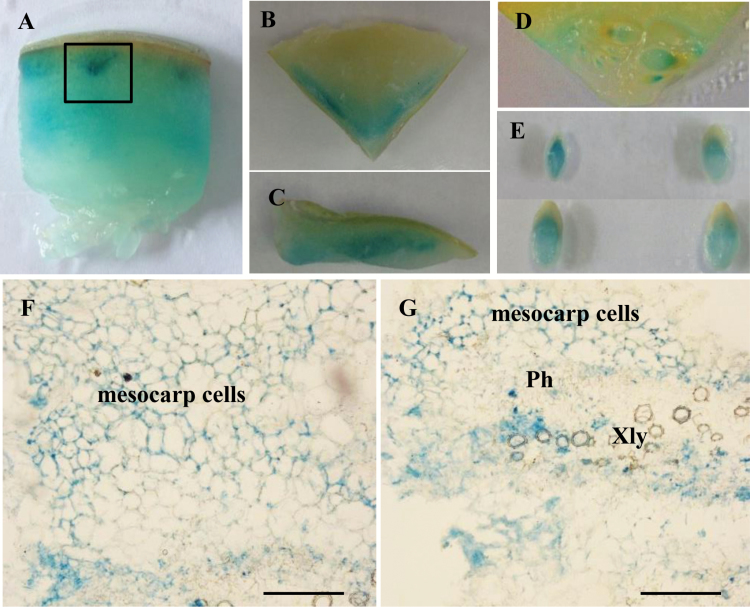
Transient expression of *CmTST2*-promoter-GUS in melon fruit. (A) A piece of fruit at 10 d after anthesis (DAA). (B) Transverse section of the top part of a fruit. (C) Vertical section of the top part of a fruit. (D) Placenta of a fruit at 5 DAA. (E) Seeds from a fruit at 10 DAA. (F, G) Paraffin sections of the fruit tissue from (A), as marked by the black box. Xyl, xylem; Ph, phloem; Scale bars =200 μm. (This figure is available in colour at *JXB* online.)

Given that *CmTST2* was the mostly highly expressed TST isoform in melon fruit, we performed histochemical and immunohistochemical analyses to determine its cell-type localization in melon fruit tissues. Transient expression of *CmTST2-promoter-GUS* in the fruit and histochemical analysis showed that GUS activity was detectable in the mesocarp and in the developing seeds from 5 or 10 DAA (Fig. 5A–E). To obtain more detailed information, we made paraffin sections and found that GUS activity could be detected in the mesocarp cells and the vascular bundles Fig. 5F, G).

**Fig. 6.**
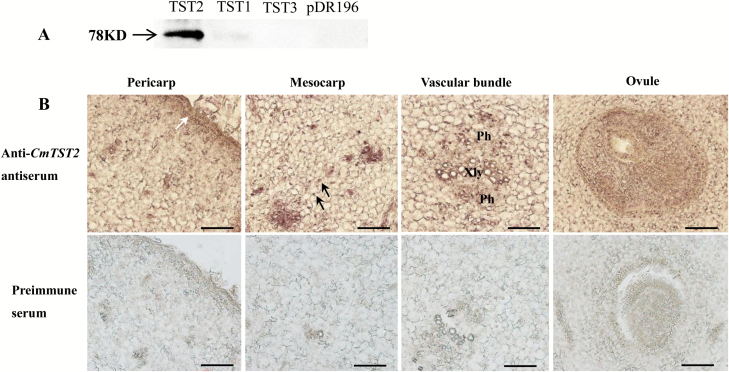
Immunolocalization of CmTST2 in melon. (A) Western blot of yeast-expressed *CmTST2* to test the specificity of the anti-CmTST2 antiserum. A specific 78-kD band was detected only in cells expressing *CmTST2* and was absent in cells expressing *CmTST1*, *CmTST3*, or the pDR196 empty vector. (B) Transverse sections of melon fruit at 0 d after anthesis. Xyl, xylem; Ph, phloem. The white arrow indicates the epicarp; black arrows indicate the mesocarp cells. Scale bars =100 μm. (This figure is available in colour at *JXB* online.)

Immunohistochemical analysis was conducted to detect the localization of the CmTST2 protein in melon fruit tissues. To test the specificity of the antisera, western blotting was performed first and the results showed that the anti-CmTST2 antiserum was selectively bound with CmTST2 (Fig. 6A). For the immunohistochemical localization, the results indicate that CmTST2 was expressed in the epicarp, mesocarp, vascular bundles, ovules, and the cells around the ovule (Fig. 6B). Labeling was absent in sections probed with the pre-immune serum. This finding was consistent with the result of the histochemical analysis of GUS expression (Fig. 5).

### Overexpression of CmTST2 can increase sugar accumulation in strawberry and cucumber fruit

**Fig. 7.**
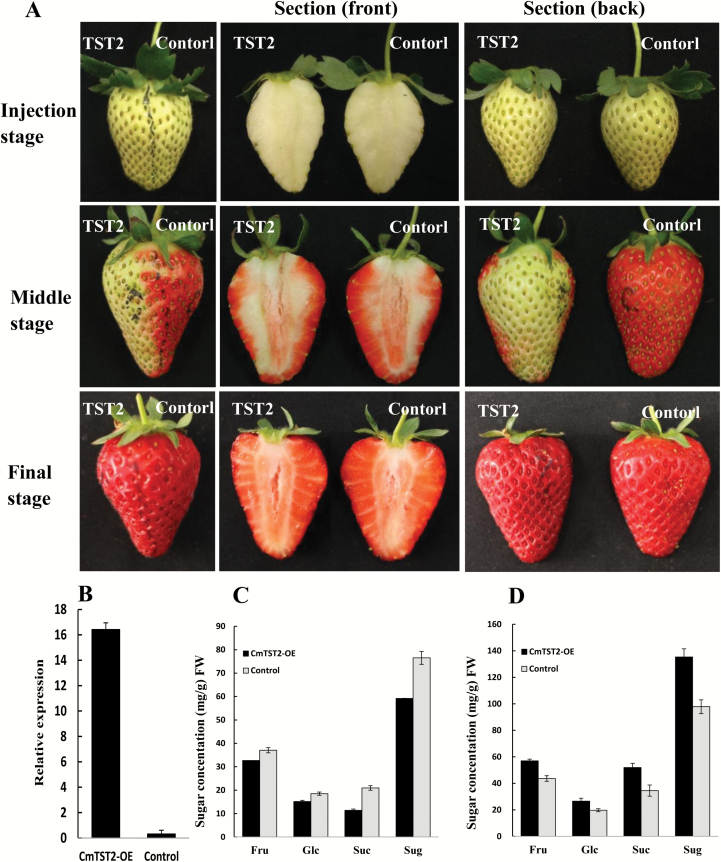
Overexpression of *CmTST2* in strawberry fruit. (A) Effect of *CmTST2* overexpression on strawberry fruit ripening (cv Falandi). (B) qRT-PCR analysis of *CmTST2* expression in the TST2 part (*CmTST2*-overexpression) and the control part of the fruit. (C) Sugar concentrations in the TST2 and control parts of the fruit at the middle stage of development. (D) Sugar concentrations in the TST2 and control parts of the fruit at the final stage of development. Sug represents the sum of the fructose, glucose, and sucrose. Values are means ±SE of four replicates.

To identify the possible role of CmTST2 in fruit development, we generated the *CmTST2* overexpression construct by cloning the coding region of *CmTST2* into the restriction site of XbaI and SmaI in the PBI121 vector under the control of the CaMV35S promoter. First, we used strawberry fruit as the transient expression system. The construct with the *CmTST2* gene and the PBI121 empty vector (control) was injected into different parts of the strawberry fruit at the late green fruit stage while the fruit was still attached to the plant. We found an interesting phenotype during fruit development: about 3–5 d after injection (middle stage), the half of the fruit that had been injected with the empty vector was nearly fully red while the part injected with *CmTST2* did not turn red (Fig. 7A). However, the *CmTST2*-injected part was also fully red and matched the control part at the final stage of development (Fig. 7A). We determined the sugar concentrations in these two halves of the fruit at the middle and final stages of development. In the *CmTST2*-overexpression half, the concentrations of fructose, glucose, sucrose, and total sugars were lower than those in the control at the middle stage (Fig. 7C). At the final stage, the contents of all sugars (fructose, glucose, and sucrose), and especially sucrose, in the *CmTST2*-overexpression half were higher than those in the control (Fig. 7D). Quantitative analysis showed that the expression of *CmTST2* in the *CmTST2*-injected half was significantly higher than that in the control (Fig. 7B). These results indicated that the overexpression of *CmTST2* in strawberry fruit delayed the fruit in turning red but increased sugar accumulation in the final stage of the fruit development.

**Fig. 8.**
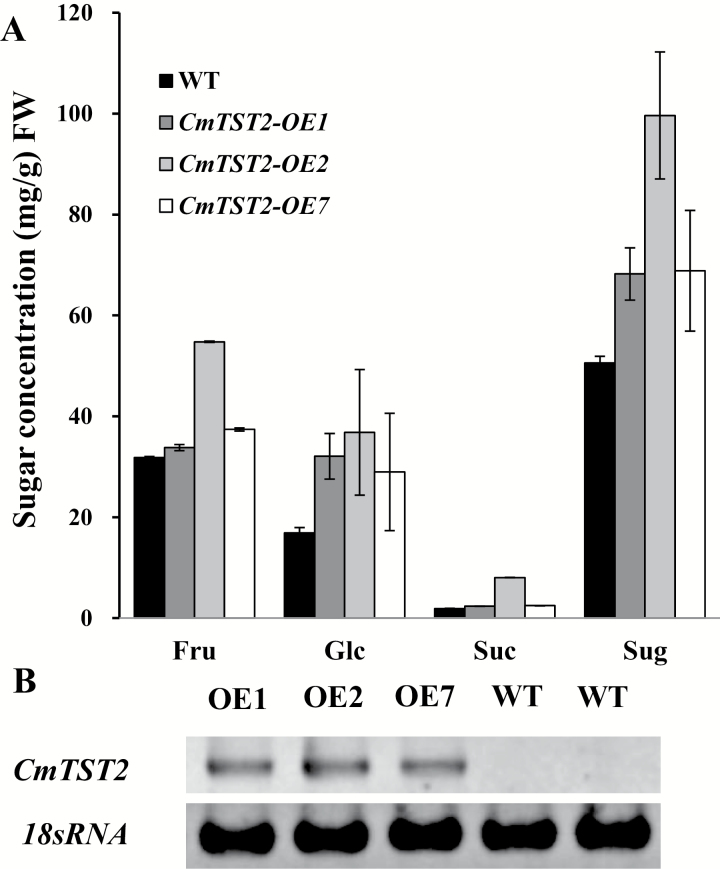
Overexpression of *CmTST2* in cucumber plants. (A) Sugar concentrations in *CmTST2* overexpression (OE) lines and wild-type cucumber fruit at 9 d after anthesis. Values are means ±SE of four replicates. (B) RT-PCR analysis of *CmTST2* expression in *CmTST2* OE and wild-type cucumber plants.

Cucumber plants, which belong to the gourd family, contain low amounts of sugar in their fruit and thus they are suitable for examining the function of CmTST2. We transformed the *CmTST2* overexpression construct into the cucumber plants and generated several overexpressing transgenic lines. Three lines showed a distinct *CmTST2* mRNA accumulation (Fig. 8B) and we used these to generate T2 generations for subsequent analyses. We determined the sugar concentrations in the fruit of these three lines (9 DAA, marketable mature fruit) and found that the contents of fructose, glucose, sucrose, and total sugar increased in the transgenic plants compared with those in the wild-type (Fig. 8A). These results demonstrated that overexpressing *CmTST2* in cucumber fruit can increase sugar accumulation.

## Discussion

### Tonoplast sugar transporters (CmTSTs) in melon

Sugar transport in plants is mediated by three transporter families: SUCs/SUTs (sucrose transporters) (Riesmeier *et al.*, 1992; Sauer, 2007; Kühn and Grof, 2010), MSTs (monosaccharide transporters) (Büttner, 2007), and SWEETs (hexose and sucrose transporters) (Chen *et al.*, 2010, 2012; Eom *et al.*, 2015). Based on their functional characterization, these transporters play different roles in plants, depending on the type of plants and tissues. In sugar-storage organs, such as sugar beet taproots, sugarcane stems, and many fleshy fruits (e.g. grape berries and melons), a major fraction of the sugars entering the cell is directed toward the vacuole, indicating that sugar transporters are needed across the vacuole membrane.

In recent years, several tonoplast-localized sugar transporters have been identified at the molecular level. Previous studies have reported that two clades from the monosaccharide transporter family, namely tonoplast monosaccharide transporters (TMTs) (Wormit *et al.*, 2006) and vacuolar glucose transporters (VGTs) (Aluri and Büttner, 2007), are exclusively localized in the tonoplast and probably act as glucose proton antiporters to facilitate uptake into the vacuole. This finding was verified for TMT1 by using electrophysiological methods (Schulz *et al.*, 2011). Another type of transporter, namely the Arabidopsis ERDL6 (early response to dehydration-like 6) protein, and its sugar beet homolog BvIMP, both act as proton-coupled vacuolar glucose exporters (Poschet *et al.*, 2011; Klemens *et al.*, 2014). Moreover, some SUC4-type sucrose transporters, such as LjSUT4 and AtSUC4, are localized to the vacuolar membrane and act as sucrose/proton symporters to facilitate the release of sucrose from the vacuole to the cytosol (Reinders *et al.*, 2008; Eom *et al.*, 2011; Schulz *et al.*, 2011; Schneider *et al.*, 2012). Furthermore, SWEET homologs 2, 16, and 17 act as vacuolar sugar facilitators, which mediate sugar export from the vacuole under *in vivo* conditions (Chardon *et al.*, 2013; Klemens *et al.*, 2013; Guo *et al.*, 2014; Chen *et al.*, 2015).

Although several tonoplast sugar transporters and facilitators have been identified, limited information is available regarding transporters involved in sucrose uptake into the vacuole. Proteomic and electrophysiological analyses have indicated that one member of the TMT homolog from sugar beet, BvTST2.1, acts as a sucrose proton antiporter for vacuolar uptake in sugar beet taproots (Jung *et al.*, 2015). BvTST2.1 is a sucrose-specific transporter that exhibits high amino-acid sequence similarity to the TMT family in Arabidopsis; as a result, this transporter family was renamed from tonoplast monosaccharide transporters (TMTs) to tonoplast sugar transporters (TSTs) (Jung *et al.*, 2015).

Melon fruit accumulate high amounts of sucrose during their development and this makes them an important horticultural cash crop; as a result, much research has focused on identifying the TST proteins involved in sugar accumulation in melons. This study reports the cloning and functional characterization of three cDNAs encoding putative TST proteins from melon plants. Phylogenetic analysis indicated that these three proteins, CmTST1, CmTST2, and CmTST3, share amino acid sequence identity with the TMT (TST) sub-family from Arabidopsis and other plants (Fig. 1). CmTST2 shares high amino acid identity (81%) with BvTST2.1, which is a newly identified vacuolar sucrose-specific transporter in sugar beet (Jung *et al.*, 2015). Similar to *BvTST2.1*, *CmTST2* is the most abundant TST gene during melon fruit development (Fig. 2) and was found to have much higher expression in a high sugar-containing variety (Yilishabai) than in low sugar-containing varieties (Xiaomaisu and Yangjiaosu) (see Supplementary Fig. S3B). Although the highest expression of *CmTST2* occurred at 20 DAA before sucrose began to be accumulated in Yilishabai, the existence of ORFs in the 5′-untranslated region of the mRNA of *CmTST2* (data not shown) indicated that it is possible that this was due to the regulation of translation. Furthermore, the CmTST2 protein was clearly located to the vacuolar membrane, as evidenced when it was expressed in tobacco epidermal cells and protoplasts (Fig. 3). Hence, CmTST2 could be mainly responsible for sucrose accumulation in melon fruit.

### CmTST2 plays an important role in sugar accumulation in fruit

Most fleshy fruits, including sweet melon, strawberry, and apple, usually accumulate high sugar contents; for example, sugar accounts for up to 16% of the fresh weight of mature fruit in sweet melon plants. In sugar-storage organs such as melon fruit, the major fraction of sucrose entering the cells is directed toward the vacuole; however, the mechanism through which sucrose is transported against an inverse concentration gradient remains unclear. The tonoplast sugar transporter BvTST2.1 is responsible for sucrose accumulation in sugar beet taproots (Jung *et al.*, 2015) But, thus far, the functions and characteristics of transporters in fleshy fruits have not yet been reported. Melon represents a typical rafffinose family oligosaccharide-translocating plant and accumulates high sugar contents in its mature fruit (Dai *et al.*, 2006, 2011). It is therefore important that the roles of these transporters in melon plants should be determined.

Three TST genes are present in the melon plant, and *CmTST2* is the most highly expressed isoform during fruit development as sugar accumulates. The histochemical and immunohistochemical analyses revealed the transcription and translation of *CmTST2* in the mesocarp cells in the fruit, which accumulate most of the sugars (Figs 5, 6). To assess the substrate specificity and physiological function of CmTST2 in living plant cells, we transiently expressed the *CmTST2* gene in strawberry fruit. We found an interesting phenotype, whereby overexpressing *CmTST2* could delay fruit ripening (turning red) in the first few days after injection with the vector and led to less sugar accumulation than in the control (Fig. 7). This finding could be due to regulation of the sucrose signal. In previous studies, the sucrose content in the cytosol has been identified as having a function as a signal involved in regulating strawberry fruit ripening (Jia *et al.*, 2013). Down-regulation of the expression levels of FaSUT1, a sucrose transporter localized to the plasma membrane in strawberry fruit, arrested fruit ripening, as reflected by the pale colour of the fruit. By contrast, overexpression of FaSUT1 accelerated fruit ripening (Jia *et al.*, 2013). Here, the overexpression of *CmTST2*, a tonoplast sugar transporter, in strawberry fruit may result in the import of more sucrose into the vacuole and may temporarily decrease the sucrose content in the cytosol before the feedback signal is adjusted, thereby inhibiting fruit ripening. During fruit development, the delayed-ripening phenotype was alleviated and disappeared following the adjustment of the signal feedback. When both the fruit with *CmTST2* overexpression and the control fruit were red and completely ripened, the tissues in the former had accumulated more fructose, glucose, and sucrose than the control (Fig. 7), which indicated that CmTST2 can transport these sugars into the vacuole. We also stably transformed the *CmTST2* gene in cucumber plants, which belong to the same gourd family as melon but contain low sugar concentrations in their fruit. The results demonstrated that overexpressing *CmTST2* could increase the accumulation of fructose, glucose, and sucrose in the cucumber fruit and confirmed that the substrates of CmTST2 may include these sugars (Fig. 8). Previous studies have shown that TMT1/2 from Arabidopsis (Wormit *et al.*, 2006; Schulz *et al.*, 2011) and TST1 from *Beta vulgaris* (Jung *et al.*, 2015) can transport not only glucose and/or fructose, but also sucrose.

### CmTST1, CmTST2, and CmTST3 play different roles in plants

Similar to the TMT protein family in Arabidopsis (Wormit *et al.*, 2006), the TST family from melon plants also comprises three isoforms. This is different from rice and sugar beet that both contain four TMT or TST isoforms (Cho *et al.*, 2010; Jung *et al.*, 2015). The different expression patterns of the three *AtTMT* genes have been studied in Arabidopsis by using promoter-GUS activity analysis (Wormit *et al.*, 2006). In Arabidopsis, *AtTMT1* showed wide expression in developing seedlings, roots, leaves, petals, filaments, and pollen cells. *AtTMT2* expression was prominent in the root stele, the edges of mature leaves, and petals and filaments of flowers. By contrast, *AtTMT3* was expressed at very low levels in all Arabidopsis tissues (Wormit *et al.*, 2006).

In this study, we also analysed the promoter activity of the three *CmTST* genes in Arabidopsis and obtained interesting results. Similar to *AtTMT1*, *CmTST1* showed a wide expression pattern, particularly in tissues and during developmental phases with high metabolic turnover, which included germinating seeds, young seedlings, and developing flower buds and filaments (Fig. 4A). In contrast with AtTMT1, which showed high expression in pollen grains, we could not detect any GUS activity in the pollen grains of *CmTST1*-promoter-GUS transgenic plants. *CmTST1* also showed high expression in wounded tissues and abscission zones. Wounding induces a wide number of cellular responses and the sealing of wound sites requires metabolic energy that is provided by the pools of soluble sugars (de Bruxelles and Roberts, 2001). A vacuolar glucose exporter, AtERDL6, has been found to show a rapid increase in expression under wounding conditions and also under treatment with methyl jasmonate, a phytohormone that regulates the response of plants to wounding (Poschet *et al.*, 2011). As a vacuolar sugar importer, the role that CmTST1 plays in this process will be a very interesting subject to investigate in the future.

Interestingly, *CmTST2*-promoter-GUS activity was mainly restricted to the vascular tissues of different organs, including the cotyledon, hypocotyl, leaves, sepals, and petals (Fig. 4B). Histochemical and immunohistochemical analyses in melon fruit tissues also showed high *CmTST2* expression in the vascular bundles in addition to the mesocarp cells (Figs 5, 6). Similar results have been found in two TMT genes from rice, *OsTMT1* and *OsTMT2*, which were mainly expressed in the vascular tissues in both the source and sink (Cho *et al.*, 2010). These results suggest that CmTST2 can not only accumulate sugars in the storage organ (melon fruit) but that it is also likely to be able to retrieve and temporally store sugars in the vacuoles of vascular tissues in other organs. Both *CmTST1* and *CmTST2* generally showed high expression in roots but only *CmTST1* showed expression in the root tip (Figs 4A, B). This finding implies that it may be involved in importing excess cytosolic sugar into vacuoles for storage in roots in a different way. Two vacuolar sugar facilitators, SWEET 16 and 17, play important roles in facilitating bidirectional fructose transport across the tonoplast of roots in response to metabolic demand in order to maintain cytosolic fructose homeostasis (Klemens *et al.*, 2013; Guo *et al.*, 2014).

*CmTST3* unexpectedly exhibited strong expression in developing pollen grains and seemed to be pollen-specific (Fig. 4C, Supplementary Fig. S5B), which is very different from *TMT3* in Arabidopsis (Wormit *et al.*, 2006). A vacuolar glucose transporter, AtVGT1, from another subfamily has been found to be mainly expressed in pollen and to affect seed germination and flowering (Aluri and Büttner, 2007). This study has found the first pollen-specific TMT/TST gene in plants, and it implies that CmTST3 may play an important role in pollen development and flowering. No GUS activity under the control of the *CmTST* promoter was found in the pods and seeds of transgenic Arabidopsis (Fig. 4). However, the expression of *CmTST2* could be strongly detected in melon fruit by qRT-PCR, promoter-GUS assays, and immunohistochemical analyses in melon fruit (Figs 3, 5, 6). The difference was probably due to because the fact that Arabidopsis produces pods whereas melon plants have fleshy fruit. Note that the expression of *CmTST1* was very high in the early stage of melon fruit development; however, it decreased quickly after 20 DAA (Fig. 2B). Correspondingly, melon fruit mainly accumulated glucose and fructose in the early stages up to 25 DAA (Fig. 2A). This finding prompted us to speculate that CmTST1 is probably responsible for the accumulation of glucose and fructose in the vacuoles of melon fruit during their early development. Moreover, we conducted a bioinformatics-driven analysis of the promoter elements of the three *CmTST* genes and found the presence of some sugar, phytohormone, and abiotic stress-responsive elements (Supplementary Table S2), which should provide us with some insights into the regulation of the individual genes of *CmTST*s.

In conclusion, we isolated and characterized the TST genes (*CmTST1*, *CmTST2*, and *CmTST3*) in melon and demonstrated the physiological roles of CmTST2 in plants. We found that it functions differently to BvTST2.1, which is a sucrose-specific vacuolar transporter responsible for sucrose accumulation in sugar beet tap roots. Our current data indicate that CmTST2 from melon can not only accumulate sucrose but also glucose and fructose in fruit. A comparative analysis of the expression patterns of *CmTST1*, *CmTST2*, and *CmTST3* revealed that they differ considerably from the *TMT*s in Arabidopsis. Thus, our data suggest that TSTs are evolutionarily conserved in many plants but are likely to have functional divergence in different plants and tissues.

## Supplementary data

Supplementary data are available at *JXB* online.

Table S1. Primers used in this study.

Table S2. Potential cis-acting regulatory elements identified in the promoter regions of *CmTST* genes.

Fig. S1. Comparison of the amino acid sequences of CmTST proteins with the previously identified tonoplast-localized sugar transporters AtTST2 and BvTST2.1.

Fig. S2. Membrane-spanning model of CmTSTs.

Fig. S3. The expression of *CmTST2* in different melon genotypes with different sugar accumulation.

Fig. S4. Subcellular localization of CmTST1/3-GFP fusion protein in tobacco.

Fig. S5. Spatial expression analysis of CmTST genes in various melon organs by qRT-PCR and RT-PCR.

Supplementary Figures S1-S5 and Table S1-S2
