# Supplementary material for: Overexpression of the tonoplast sugar transporter CmTST2 in melon fruit increases sugar accumulation
Source: J Exp Bot. 2017 Dec 23;69(3):511–23. doi: 10.1093/jxb/erx440 (PMC5853577; doi:10.1093/jxb/erx440)
Supplement: Supplementary Figures S1-S5 and Table S1-S2 [file erx440_suppl_supplementary_figures_s1-s5_and_table_s1-s2.pdf]

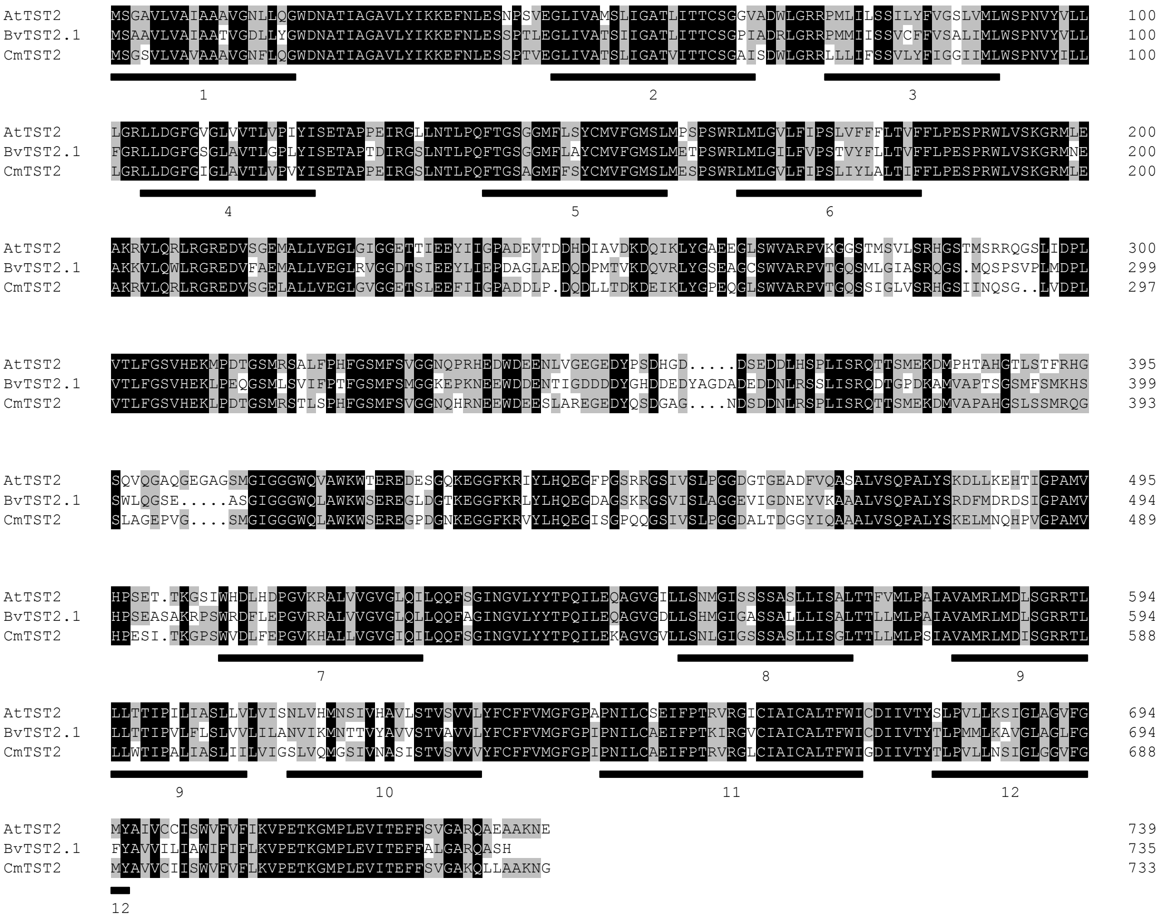

Supplementary Fig. S1. Comparison of amino acid sequence of CmTST proteins and previously identified tonoplast localized sugar transporter AtTST2 (Arabidopsis) and BvTST2.1 (sugar beet). Black boxes indicate identical amino acid residues. Dark gray boxes represent amino acid residues with an identity of more than 50%. The 12 putative trans-membrane domains of CmTST2 are underlined. The sequence alignment was performed using the DNAMAN software package.

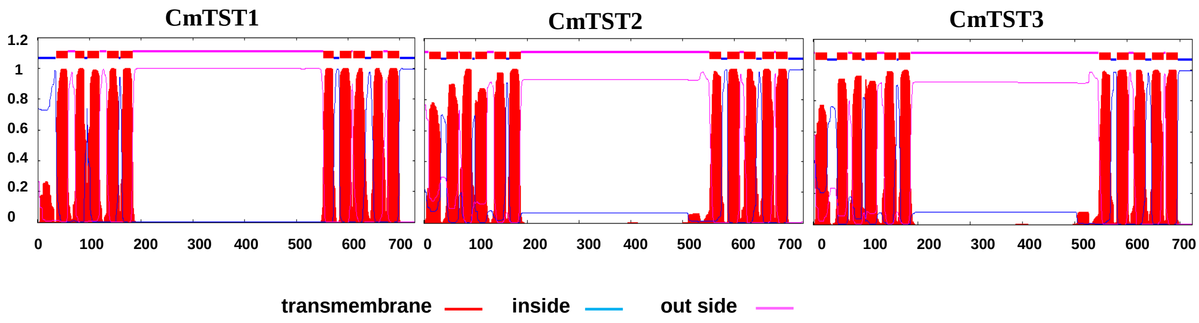

Supplementary Fig. S2. Membrane-spanning model of CmTSTs. The predicted website was TMHMM http://genome.cbs.dtu.dk/services/TMHMM-2.0.

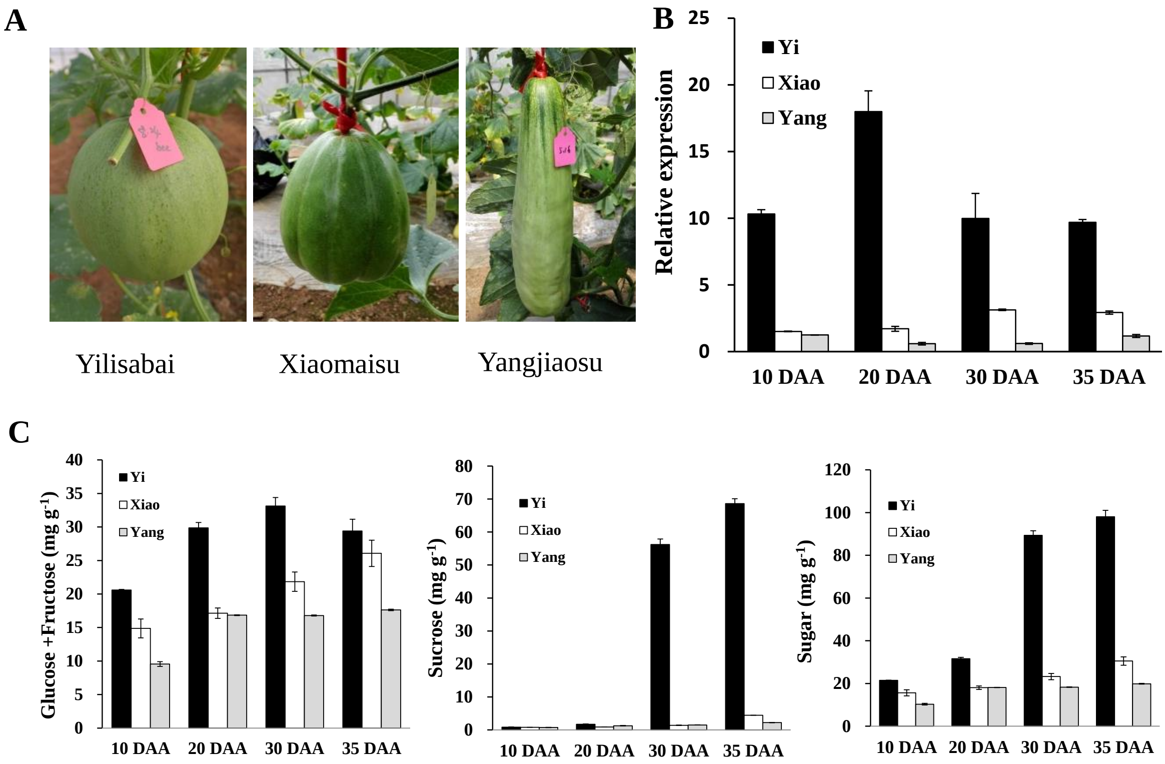

Supplementary Fig. S3. The expression of *CmTST2* in different melon genotypes with different sugar accumulation. (A) The fruit appearances of the three melon varieties, Yilisabai (Yi), Xiaomaisu (Xiao), and Yangjiaosu (Yang). (B) Relative expression of *CmTST2* in the melon varieties Yi, Xiao and Yang during different developmental stages were determined by qRT-PCR. *TUA* was used as reference gene. (C) Different types of sugar content in the three melon varieties' fruits during different developmental stages were determined by GC-MS. 'Sugar' means the total of glucose, fructose and sucrose. Error bars represent the SE for three technical replicates of three biological replicates.

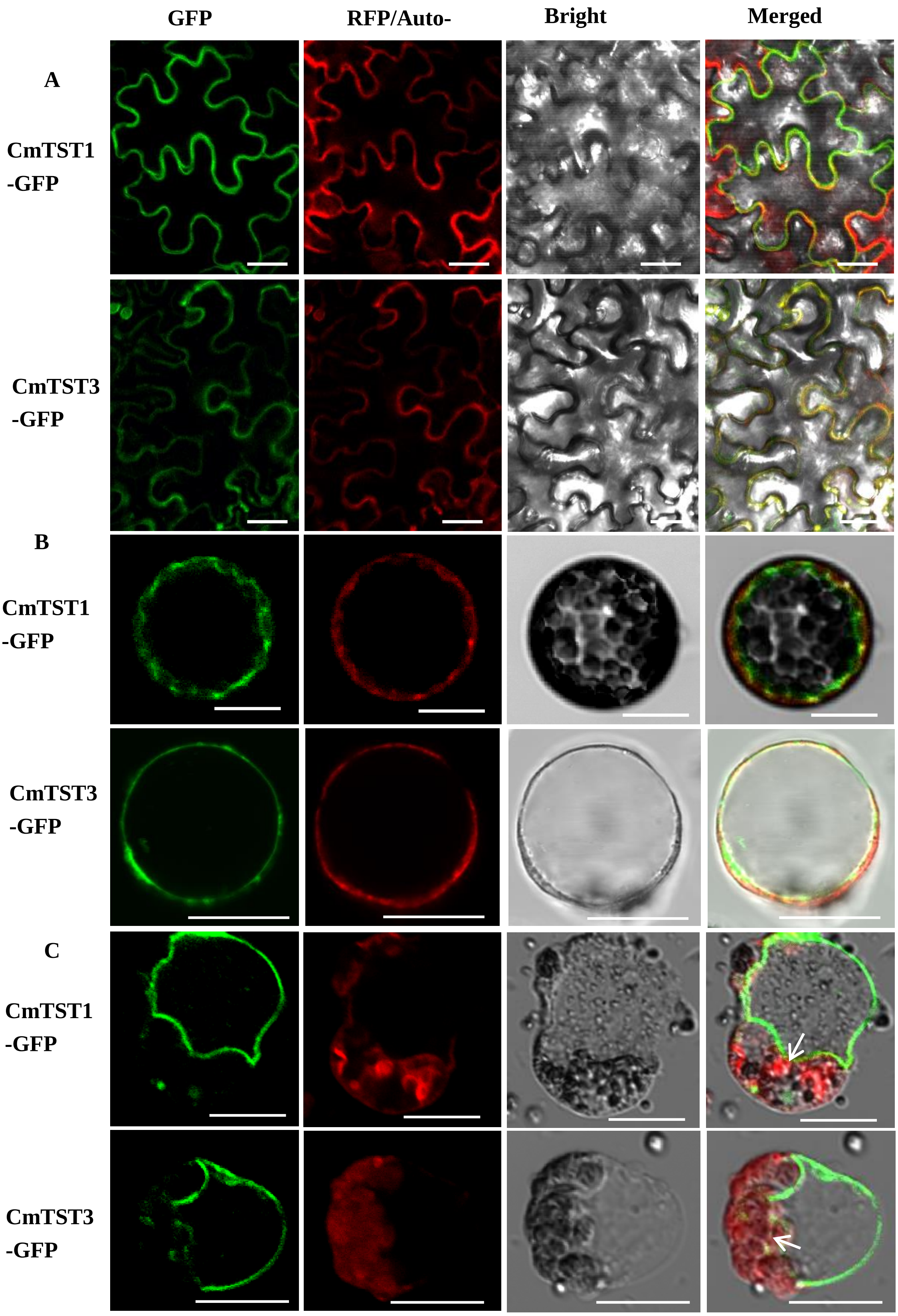

Supplementary Fig. S4. Subcellular localization of CmTST1/3-GFP fusion protein in tobacco. (A, B) Colocalization of CmTST1/3-GFP and AtRtip-RFP (tonoplast localization marker) in tobacco epidermal cell (A) or protoplast (B). (C) Tonoplast localization of CmTST1/3-GFP in tobacco protoplast. The laser-scanning confocal microscopy images show fluorescence and merged images. The chlorophyll auto fluorescence (Auto-) in (C) and the bright field images are also presented. CmTST1/3-GFP fusion protein is clearly inserted into the tonoplast, which separates the vacuolar volume from the cytoplasm containing chloroplasts (white arrows). Bars=20 μm.

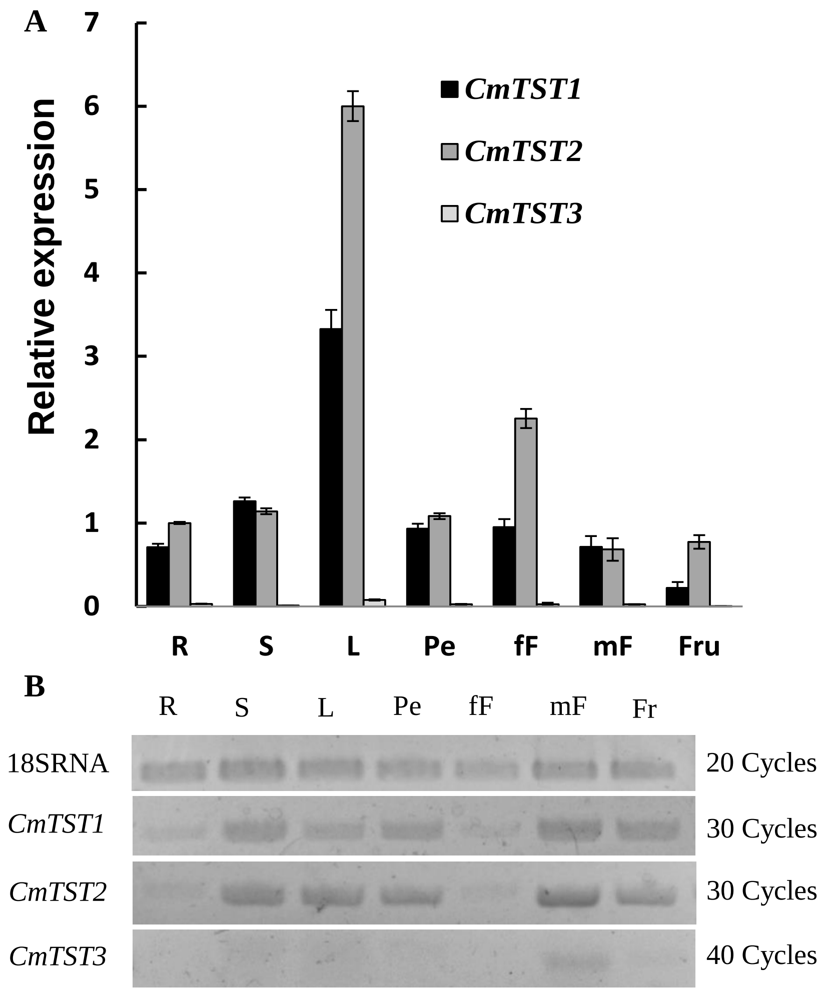

Supplementary Fig. S5. Spatial expression analysis of *CmTST* genes in various melon organs by qRT-PCR (A) and RT-PCR (B). R, root; S, stem; L, leaf; Pe, petiole; fF, female flower; mF, male flower; Fr, fruit (20 DAA). Error bars represent the SE for the three technical replicates of the three biological replicates.

Supplementary Table S1. Primers used in the paper (Lowercases indicate the restriction enzyme sites or recombination connector sequence).

primers	*Description*	*Sequence (5'- -3')*
TST1FLF	ORF clone	ATGAAGGGAGCTTTGCTAGTGGC
TST1FLR	ORF clone	TCAATTAATACTACCTTTCGCAGCT
TST2FLF	ORF clone	ATGAGTGGTTCTGTTTTGGTGGCTG
TST2FLR	ORF clone	TCAACCATTTTTAGCAGCTAAAAGCTGTTTGG
TST3FLF	ORF clone	ATGAGGGGAGCTGTTCTCGTTGCAATCGC
TST3FLR	ORF clone	TTAATCAGCATTATCAGCCTGTTTCGC
TubulinF	Quantitative RT-PCR	GCGGTGCTTCTAGACAATGA
TubulinR	Quantitative RT-PCR	CCTGAGATACAAGACGGTTGAG
TST1qF	Quantitative RT-PCR	ATAATAGGTCCTGCTGAAGA
TST1qR	Quantitative RT-PCR	AAAGGGTGACAAGTGGGT
TST2qF	Quantitative RT-PCR	GGATGGGATAATGCGACTA
TST2qR	Quantitative RT-PCR	GCTCCGATAAGGGATGTG
TST3qF	Quantitative RT-PCR	GGTCGTCGTCCTCTAATG
TST3qR	Quantitative RT-PCR	ACCAAGGTAACCGCAAGT
Cm18SF	Semi RT-PCR	CGAGTCTGGTAATTGGAATGAGTA
Cm18SR	Semi RT-PCR	CTACGAGCTTTTTAACTGCAACAA
TST2F	Semi RT-PCR	AAGGATGGGATAATGCGACTA
TST2R	Semi RT-PCR	TGACAAGCCAGCGAGGTGA
TST1F	Semi RT-PCR	GCATCAGAGGCTGGTGGAG
TST1R	Semi RT-PCR	TAGCACAGGGATGGTAGCG
TST3F	Semi RT-PCR	GTCAGCCAATCAGCACTCC
TST3R	Semi RT-PCR	AGGCACCAAAGACTCCACC
TST1GFPF	Fusion to GFP	attacgccgaggtcATGAAGGGAGCTTTGCTAGTGGC
TST1GFPR	Fusion to GFP	tagggaagaggTCAATTAATACTACCTTTCGCAGCT
TST2GFPF	Fusion to GFP	attacgccgaggtcATGAGTGGTTCTGTTTTGGTGGCTG
TST2GFPR	Fusion to GFP	tagggaagaggTCAACCATTTTTAGCAGCTAAAAGCTGTTTGG
TST3GFPF	Fusion to GFP	attacgccgaggtcATGAGGGGAGCTGTTCTCGTTGCAATCGC
TST3GFPR	Fusion to GFP	tagggaagaggTTAATCAGCATTATCAGCCTGTTTCGC
TST1PF	Promoter clone	gaccatgattacgccaagcttGTCATATACTTACTTTAGCTCAACCTC
TST1PR	Promoter clone	ggactgaccacccggggatccTTATTCAATTCAAGCTCCAATGTGGTCAG
TST2PF	Promoter clone	gaccatgattacgccaagcttCAAAGTCGTTTAGATTTAGTTACCC
TST2PR	Promoter clone	ggactgaccacccggggatccTTTGCTTCTTCAAATCGACAGGAAA
TST3PF	Promoter clone	gaccatgattacgccaagcttCTTATTGTGCATCACAGCATAGAGG
TST3PR	Promoter clone	ggactgaccacccggggatccCTTACCAATGGGGTGAATTAGAATATC
TST2-121F	Clone in to PBI121	tgctctagaATGAGTGGTTCTGTTTTGGTGGCTG
TST2-121R	Clone in to PBI121	tcccccggGTCAACCATTTTTAGCAGCTAAAAGCTGTTTGG

Supplementary Table S2. Potential cis-acting regulatory elements identified in promoter regions of *CmTST* genes.

Gene name	Tissue/organ specific relative elements	Sugar / hormone responsive elements	Abiotic stress-responsive elements
CmTST1	**CAATBOX1** (CAAT, tissue-specific), **ROOTMOTIFTAPOX1** (ATATT, organ-specific), **RAV1AAT** (CAACA, high express in rosette leaves and roots), **OSE1ROOTNODULE** (AAAGAT, organ-specific) **OSE2ROOTNODULE** (CTCTT, organ-specific)	**WBOXHVISO1** (TGACT, sugar-responsive), **SREATMSD** (TTATCC, sugar-responsive), **PYRIMIDINEBOXOSRAMY1A** (CCTTTT, gibberellin-responsive), **P-box** (CCTTTTG, gibberellin-responsive) **ARFAT** (ACTTTA, auxin-responsive), **ERE** (ATTTCAAA, ethylene-responsive)	**ELRECOREPCRP1** (TTGACC, pathogen- and wound-induced), **TC-rich** (ATTTTCTTCA, defense and stress responsiveness)
CmTST2	**CAATBOX1** (CAAT, tissue-specific), **ROOTMOTIFTAPOX1** (ATATT, organ-specific), **RAV1AAT** (CAACA, high express in rosette leaves and roots), **RAV1BAT** (CACCTG, high express in rosette leaves and roots), **OSE2ROOTNODULE** (CTCTT, organ-specific), **RHERPATEXPA7** (KCACGW, root hair-specific)	**WBOXHVISO1** (TGACT, sugar-responsive), **PYRIMIDINEBOXOSRAMY1A** (CCTTTT, gibberellin-responsive), **GARE** (AAACAGA, gibberellin-responsive) **ARFAT** (ACTTTA, auxin-responsive), **DPBFCOREDCDC3** (ACACNNG, ABA-responsive), **TCA-element** (CAGAAAAGGA, salicylic acid-responsive),	**TC-rich** (ATTTTCTTCA, defense and stress responsiveness)
CmTST3	**POLLEN1LELAT52** (AGAAA, pollen-specific motif) **CAATBOX1** (CAAT, tissue-specific), **ROOTMOTIFTAPOX1** (ATATT, organ-specific)	**SREATMSD** (TTATCC, sugar-responsive), **PYRIMIDINEBOXOSRAMY1A** (CCTTTT, gibberellin-responsive), **GARE** (AAACAGA, gibberellin-responsive) **ERE** (ATTTCAAA, ethylene-responsive),	**TC-rich** (ATTTTCTTCA, defense and stress responsiveness)
